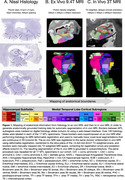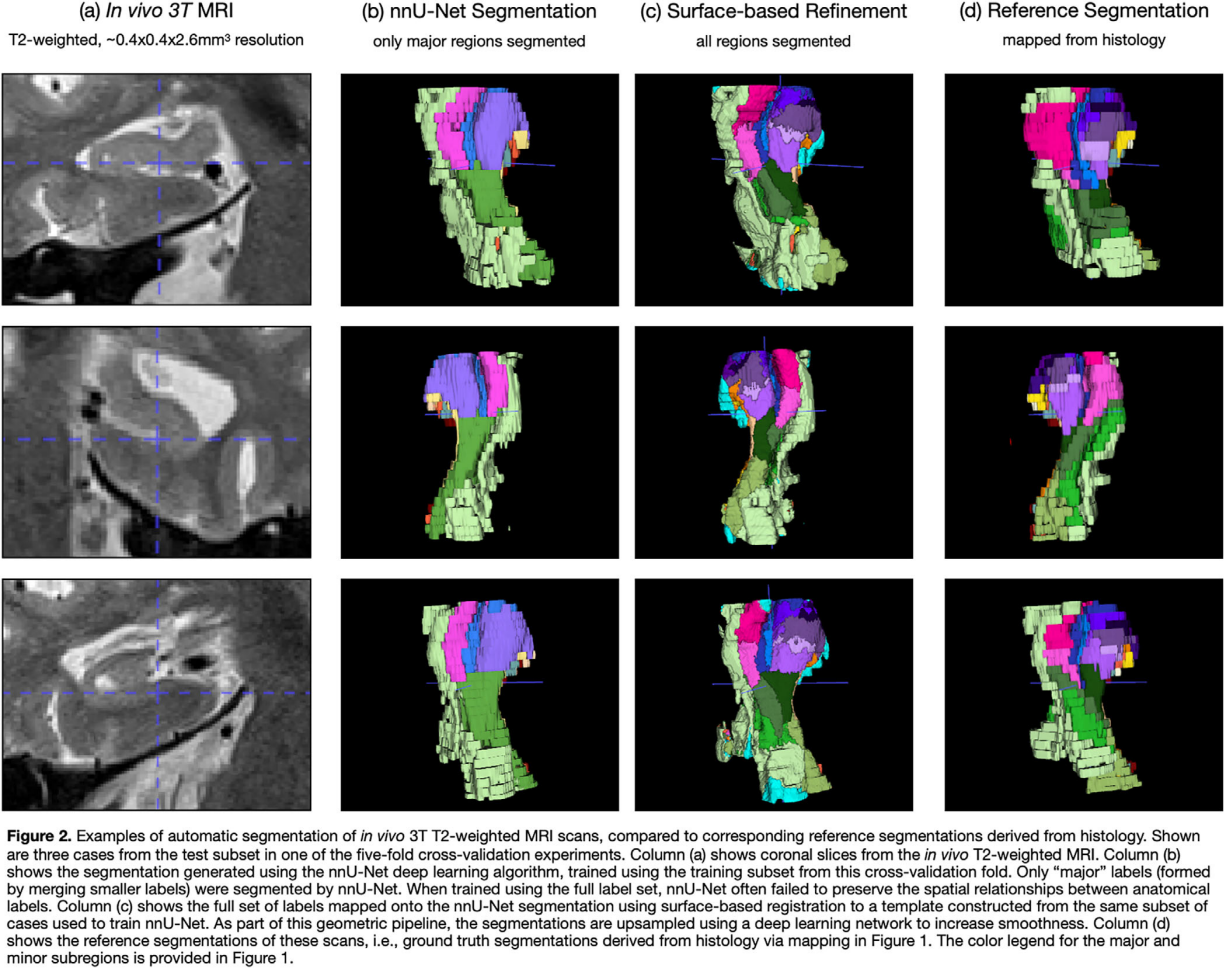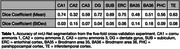# Mapping Anatomical Boundaries from Histology to Antemortem MRI to Inform Automatic Segmentation of Medial Temporal Lobe Subregions

**DOI:** 10.1002/alz.094008

**Published:** 2025-01-09

**Authors:** Amanda E Denning, Sydney A Lim, Yue Li, Niyousha Sadeghpour, Sadhana Ravikumar, Ranjit Ittyerah, Eunice Chung, Madigan Bedard, Karthik Prabhakaran, Winifred Trotman, Alejandra Bahena, Daniel T Ohm, John L. Robinson, Theresa Schuck, Dylan M Tisdall, Emilio Artacho‐Perula, Maria Mercedes Iniguez de Onzono Martin, Monica Munoz, Francisco Javier Molina Romero, Jose Carlos Delgado Gonzalez, Maria del Mar Arroyo Jimenez, Maria del Pilar Marcos Rabal, Sandra Cebada Sanchez, Carlos de la Rosa Prieto, Marta Córcoles Parada, David J Irwin, Eddie B Lee, Sandhitsu R. Das, David A Wolk, Paul A. Yushkevich, Ricardo Insausti, Laura E.M. Wisse

**Affiliations:** ^1^ University of Pennsylvania, Philadelphia, PA USA; ^2^ University of Castilla‐La Mancha, Albacete Spain; ^3^ Department of Clinical Sciences Lund, Lund University, Lund, Lund Sweden

## Abstract

**Background:**

The medial temporal lobe (MTL) is the epicenter of both primary and concomitant molecular pathologies in Alzheimer’s disease (AD). The intricate anatomy of the MTL has been the subject of extensive study over the past two centuries. However, current PET and MRI AD biomarkers use often crude parcellations of the MTL that have not been sufficiently validated vis‐à‐vis anatomical ground truth. Here we use a unique dataset of finely annotated serial histology, ex vivo MRI and antemortem 3T in vivo MRI of the MTL from 17 brain donors to train and evaluate an automatic MTL subregion segmentation algorithm. To our knowledge, this is the most comprehensive attempt to infuse MRI biomarkers with cytoarchitectural reference annotations.

**Method:**

A team of neuroanatomists annotated the boundaries of 27 MTL subregions on over 2000 serial Nissl histological sections from 17 brain donors. These boundaries were mapped to 9.4T ex vivo MRI (proton density, 0.2×0.2×0.2mm3) and, subsequently, to 3T in vivo MRI (T2‐weighted, ∼0.4×0.4×2.6mm3) using deformable registration, with extensive manual editing after each registration step to correct for registration errors and ensure 3D continuity and smoothness (Figure 1). Deep learning method nnU‐Net was trained on the resulting in vivo MRI annotations to automatically segment a set of major subregions (formed by merging smaller subregions). Surface‐based registration between an MTL template and the output of nnU‐Net was used to parcellate major subregions into 27 smaller subregions. Segmentation accuracy was assessed by five‐fold cross‐validation.

**Result:**

Figure 2 compares nnU‐Net and surface‐based automatic segmentations with corresponding cytoarchitectural reference segmentations. Table 1 reports segmentation accuracy for major subregions in terms of Dice coefficient. While accuracy is not as high as in some previous MTL subregion segmentation approaches, this is not surprising because the anatomical variability of cytoarchitecture‐based ground truth annotations in our approach is likely much higher than in prior approaches where ground truth was generated by applying heuristic/geometric rules.

**Conclusion:**

It is feasible to leverage cytoarchitecturally defined anatomical boundaries for automatic in vivo MRI segmentation. However, high variability in the location of cytoarchitectural borders poses clear limitations on MTL segmentation accuracy.